# Blood Poisoning

**Published:** 1887-09

**Authors:** Edward B. Stevens


### Blood Poisoning.

(A Paper read before the Lebanon, O., Medical Society, May 31, 1887.)

BY EDWARD B. STEVENS, M.D.

In a report that I wish to make to the society of some cases which have come under my observation, one of them a recent and fatal one, I use the expression "blood poisoning" advisedly as being more general in its significance, and for this and other reasons better adapted to my purpose than the more precise terms septicæmia, pyæmia, or other words of definite accepted meaning.

I premise what I have to say by reminding medical gentlemen of the often remarked and remarkable differences in the immunity and the susceptibility of individuals to serious effects from trivial accidents. For example, some months ago a butcher residing in this village was at work in his slaughter house in his bare feet; for some reason he suddenly stepped downward a distance of a foot or more into what he supposed was simply mud; there proved to be a plank under the mud, pierced with a tenpenny nail, upon which he landed with his entire weight, the nail passing entirely through the foot, the foot being withdraw from the nail by main force. The wound produced no unpleasant effects whatever in any respect.

On the other hand, a gentleman in the vicinity of Cincinnati last autumn stepped on a small nail slightly projecting from a board; his foot was protected by sock and slipper, so that the wound, between his toes, was a slight one indeed. There was trifling soreness for two or three days, at which time the first symptoms of tetanus were manifest, when I was sent for to be associated with a very intelligent physician who was family physician in charge. But little impression was made upon the tetanic condition, and the gentleman died about the eighth day after the accident.

All of which seems to have a direct bearing upon the vague and uncertain circumstances which develop a condition of blood poisoning. This is very well expressed by Bryant in his excellent work on surgery. He says, speaking of blood poisoning: "As to the exciting cause of the disease nothing is known. It attacks the healthy as well as the cachetic, those surrounded by perfect hygienic influences as well as those subjected to the most unfavorable, and it is found in private as well as public practice."

On the evening of Saturday, February 19th last, a young lady, clerk in a dry goods store here, very slightly pricked the ball of her thumb with a needle. It gave her no uneasiness or pain at the time, but twenty-four hours afterward pain and inflammation set in, for which I was consulted at my office the next day; but I did not see the patient until one week after the original trivial injury. At this time I found her suffering pain of the most excruciating character; the hand and arm to the elbow greatly swollen and inflamed, a broad, angry, purplish-red stripe running from the hand up the inner face of the arm nearly to the shoulder; no other constitutional manifestations; no delirium at any time. Large doses of opiates produced but little appreciable relief. Several days after my visit, that is, about ten days after the needle prick, a large blister appeared on the inside of the thumb —not an abscess proper—from which was discharged several tablespoonfuls of fluid, mostly serous but partly purulent; the discharge rapidly changed to a more decidedly purulent character, and continued from the surface of the tissues. Soon death and sloughing of the muscles commenced and continued to progress until most of the muscular tissues beneath and in front of the thumb had disappeared, leaving a cavity into which might be placed a moderate sized man's thumb. This cavity gradually filled up with a deposit of new muscle, and the resulting deformity is slight, there being at the present some stiffness of the thumb and first finger.

I might also remark that the severe pain almost entirely ceased with the beginning of the purulent formation and discharge.

Dr. Henderson, of Oregonia, in this county, relates to me a very similar case, in which his own father was the patient. The palmar surface of his hand was slightly scratched with the sharp edge of a chisel. A like train of symptoms was speedily established as in the case I have just related—additionally there was delirium for some time; finally, as in my case, there was suppuration, with gangrenous sloughing of all the muscular tissues of the palmar surface of the hand, resulting eventually in entire recovery.

On the 6th of this month (May, 1887), Mr. Monfort, of Lebanon, occupation teamster, age 26, general health supposed to be good, was whittling and by accident the penknife slipped from his hand and the point of the blade striking the outside of the calf, a penetrating wound was made to the depth of half an inch. Shortly after he reported to my office, but finding he had closed the wound with a plaster and bandage, I directed him to go home, abstain from work for a few days and keep quiet.

On the 9th I was called to see him. He had obeyed my injunction so far as to go to plowing and continued till noon of this first day's visit, when he was compelled to desist from pain. I found him suffering severe headache and great pain in the leg, which was already swollen and assuming an erysipelatous blush. The swelling rapidly increased, with continued pain, swelling extending up the limb above the knee, a dark red stripe running up the inside of the leg to the body. Delirious from about the fifth day after the accident until death, but not violent; his attention being excited, the mental condition was apparently rational, but readily lapsing into delirium; the pulse, however, remained at 80 until two days before death; appetite capricious, at times quite good, then for a day the patient scarcely taking any food.

About the 17th I found an abscess had formed at a point nearly opposite the original wound, from which several ounces of fluid, partly purulent but serous, was discharged. Shortly after pus began to discharge from the wound in abundance and continued. At the time I opened the abscess there were two or three dark spots like slight bruises on the surface of the calf; within twenty-four hours these spread so as to make a continuous black surface, which my entire hand would just about cover. This gangrenous surface did not extend, but exhibited the disposition to a line of separation, and at the time of death the sloughing process had just commenced.

From May 20th approaching dissolution rapidly progressed —driving horses, going to the river and canal, picking at the bed-clothes, pulse rapid and irregular, difficulty in swallowing, largely as if he got material in his mouth, whether fluid or solid, and did not know what to do with it. He died on the 23d, the 18th day after the wound.

After the death of Monfort I learned that he was engaged a good deal of the time during the winter and spring in hauling off hogs dead from cholera. It is probable that the continual inhalation of the exhalations of cholera hogs would so infect the system as to be ready by a slight accident or wound to invite an invasion of this sort.

At any rate, there is but little to comfort one, in the history of cases of this sort, within our own experience or the information obtained from authority. Bryant, already quoted, says: "It seems reasonable to believe that a patient may die in two or three days after the first appearance of the symptoms, and as a rule bad cases terminate during the second week."

---

A German, according to an exchange, had run up a large doctor's bill and called to see the physician concerning it, whereupon the following conversation ensued:

"Goot mornin', doctor."

"Good morning, Mr. Mosenthal. How is your family?"

"Pretty vell; I gome to ask sometings aboudt mein bill."

"Yes?" with animation.

"I don'd haf got mooch money. I vants to know vill you tage id oud in der trade?"

"I guess we can arrange that," cheerfully; "what's your business?"

"Vell—I blay me a leedle on dot trombone; undt I serenadt you more as twendy times."

